# Depression status in patient with adolescent idiopathic scoliosis: A comparative study

**DOI:** 10.1186/1748-7161-8-S1-P7

**Published:** 2013-06-03

**Authors:** T Kuru, H Yilmaz, E Dereli, F Hozatlioglu, B Çelik, I Çolak

**Affiliations:** 1 Istanbul University, Faculty of Health Science, Department of Physiotherapy and Rehabilitation, Istanbul, Turkey; 2 Haliç University, Halic University, School of Health Sciences, Department of Physiotherapy and Rehabilitation, Istanbul, Turkey; 3 Istanbul Bilgi University, School of Health Science, Department of Physiotherapy and Rehabilitation, Istanbul, Turkey; 4 Dr.Lütfi Kırdar Kartal Education and Research Hospital, Istanbul, Turkey

## Background

The adolescent years are one of the most stressful times in a person's life and if they have idiopathic scoliosis, spinal deformity and brace treatment can cause psychological effects.

## Aim

Our aim was to investigate depressive symptoms in children and adolescents with idiopathic scoliosis and their association with demographic and wearing brace variables.

## Methods

Study included 47 (7 male, 40 female) participants, 23 were using brace (mean age: 13.34±1.61) and 24 were doing 3D- Schroth method exercises (mean age:13.66±1.76) for treatment. In the brace group, mean Cobb angle was 38.30±10.70 (range:20-60), mean ATR was 12.60±5.41(range:3-25), in the exercise group mean Cobb angle was 32.16±11.62 (range:15-60), mean ATR was 8.45±4.04 (range:3-18). Both of the groups were receiving treatment for 6 months at least. Depressive symptoms in youth were assessed with the Children’s Depression Inventory (CDI), a self-report questionnaire consisting of 27 items. The CDI has wide use across chronic health conditions. A score of 13 is indicative of elevated depressive symptoms.

## Results

In the brace group 5 of 23 (21.5%) and in the exercise group 4 of 24 (16.8%) patients were scored on the CDI at or above the clinical cutoff (p=0.554). There were no significant correlation between the Cobb angle, rotation angle, BMI, age and CDI scores (p=0.214, p=0.034, p=0.335, p=0.027).

## Conclusion

Findings indicated that nearly one in five youth with adolescent idiopathic scoliosis met the clinical cutoff for depression by their own report. Study results showed that there are no significant differences between brace and exercise therapies on depression status. Multidisciplinary scoliosis teams are in an ideal position to offer early identification and optimum treatment for adolescent idiopathic scoliosis.
